# Evaluating diagnostic yield and accuracy as key performance metrics in pulmonary lung lesions

**DOI:** 10.3389/fmed.2025.1572779

**Published:** 2025-05-07

**Authors:** Junsu Choe, Hyunseung Nam, Hwan-ho Cho, Sun Hye Shin, Byeong-Ho Jeong, Sang-Won Um, Hojoong Kim, Kyungjong Lee

**Affiliations:** ^1^Division of Pulmonary and Critical Care Medicine, Department of Medicine, Samsung Medical Center, Sungkyunkwan University School of Medicine, Seoul, Republic of Korea; ^2^Department of Electronics Engineering, Incheon National University, Incheon, Republic of Korea

**Keywords:** diagnostic yields, radial endobronchial ultrasound, lung nodules, navigation bronchoscopy, tissue diagnosis

## Abstract

**Objective:**

A conservative definition of diagnostic yields for assessing the performance of guided bronchoscopy has been proposed, but it has yet to be validated in practice.

**Methods:**

Patients who underwent radial endobronchial ultrasound (R-EBUS) between April 2020 and April 2023 were included in the study. Diagnostic results were classified as malignant or non-malignant based on the post-lung-biopsy pathology. Non-malignant results were further categorized into specific benign (SB), nonspecific benign (NSB), atypical cells, and non-diagnostic (ND). All non-malignant lesions were confirmed using alternative biopsy methods or chest computed tomography (CT) during a follow-up of over 1 year. Diagnostic yield and accuracy were calculated using pre-defined methods (Box below). Predictors of sampling success were identified in a logistic regression analysis.

**Results:**

Among the 736 patients evaluated in this study, R-EBUS-guided TBLB revealed malignancy in 431 (58.6%) patients. The remaining 305 (41.4%) patients with non-malignant lesions were classified as SB (8.3%), NSB (21.3%), atypia (4.6%), and ND (7.2%). Diagnostic yield vs. accuracy values based on conservative, intermediate, and liberal definitions were 67% vs. 67, 88% vs. 77, and 100% vs. 79%, respectively. Thus, for the conservative definition, diagnostic accuracy and diagnostic yield were identical. Significant predictive factors for successful lung biopsy according to the conservative diagnostic yield included lesion size (> 20 mm), CT-bronchus subclassification (Ia, Ib), and radial probe position within the lesion.

**Conclusion:**

Our study validated the use of the conservative definition of diagnostic yield as a reliable diagnostic endpoint for evaluating the performance of guided bronchoscopy. This definition could serve as a time-saving standard in prospective studies comparing the diagnostic effectiveness of various navigation devices.

## Introduction

The landscape of pulmonary diagnostics has evolved significantly, particularly the improved detection of pulmonary nodules and lesions on chest computed tomography (CT) scans (1, 2). Such findings, whether incidental or part of a screening program, raise suspicion of early stage lung cancer as well as other critical pathologies, making timely and accurate biopsy procedures vital (3).

In this context, navigation bronchoscopy has become a key approach in accessing peripheral pulmonary lesions that are often difficult to reach using traditional bronchoscopy techniques. Radial endobronchial ultrasound (R-EBUS)-guided transbronchial lung biopsy (TBLB), a foundational method within this domain (4), offers a minimally invasive, safe, and efficient approach to sampling lung lesions, with a complication rate lower than that of other, more invasive, procedures (5). Alongside R-EBUS, newer techniques such as electromagnetic navigation bronchoscopy and robotic bronchoscopy provide enhanced navigation to peripheral lesions and have thus gained traction in clinical practice (6). The two methods offer distinct advantages: electromagnetic navigation excels in mapping and directing biopsy tools to precise locations, while robotic bronchoscopy offers greater control and reach. The choice often depends on institutional resources, operator expertise, patient preference, and cost-effectiveness considerations (7).

The rapid development and adoption of advanced navigation bronchoscopy have led to an increased focus on defining robust performance metrics for these diagnostic techniques (8). Traditionally, diagnostic yield (the percentage of procedures that successfully lead to a diagnosis) and diagnostic accuracy (how well a test provides the correct diagnosis) have been the primary benchmarks for evaluating bronchoscopic procedures. However, the complexity of pulmonary nodules, which can encompass both malignant and benign pathologies, complicates the use of these traditional metrics. In response, the American Thoracic Society/American College of Chest Physicians has proposed a standard metric based on Delphi consensus guidelines (9). The “conservative diagnostic yield” limits the numerator in the diagnostic yield calculation to cases in which the biopsy yields either malignant or specific benign findings that are clinically actionable. With its focus on results that are directly relevant to patient management, this metric provides a clear indication of procedural success. An early report showed that the conservative yield differs by only ~1% from the traditional diagnostic accuracy and therefore that it aligns closely with accuracy while offering a more focused measure of clinical utility (10).

Given the potential implications of adopting the conservative diagnostic yield as a standard metric, especially in clinical practice and research, its utility in real-world settings must be validated.

**Table:** 

1.Conservative diagnostic yield = (Malignant results + Specific benign results)/Total procedures. 2.Intermediate diagnostic yield = (Malignant results + Specific benign results + Non-specific benign results)/Total procedures. 3.Liberal diagnostic yield = (Malignant results + Specific benign results + Non-specific benign results + Atypical + Non-diagnostic results)/Total procedures.

Thus, the aim of this study was to evaluate the performance of R-EBUS-guided TBLB using a range of diagnostic yield definitions, from conservative to liberal, and to compare diagnostic accuracy when using these definitions. The impact of the tissue diagnosis on subsequent patient-management decisions was also examined. Our results provide insight into how different diagnostic metrics influence clinical decisions, such as the need for additional treatments or invasive procedures.

## Materials and methods

### Study design and participants

This was a single-center, retrospective cohort analysis utilizing the R-EBUS database. We included all patients who underwent R-EBUS-guided transbronchial lung biopsy (TBLB) at our institution between April 2020 and April 2023 for lesions or nodules requiring further characterization. For patients with pathological results that were inconclusive or for whom no further treatment was deemed necessary based on clinical features, follow-up of at least 1 year was required. Patients lost to follow-up after lung biopsy or who underwent the procedure to confirm disease recurrence or for genetic studies were excluded. This study was approved by the Institutional Review Board (SMC 2023-10-083) of Samsung Medical Center. The requirement for informed consent was waived due to the retrospective nature of the study.

### Radial EBUS guided lung biopsy as the index test

Radial EBUS-guided lung biopsy served as the index test in patients with abnormal findings detected on chest CT scans. The decision to proceed with radial EBUS-guided biopsy was made at the physician’s discretion. Prior to the procedure, all patients’ chest CT scans were reviewed. A radial probe inserted through the bronchial tree using a thin 4 mm bronchoscope was used to perform TBLB on the identified lung lesion. All procedures were conducted with the patient under conscious sedation with intravenous midazolam and fentanyl. Following the completion of TBLB, specific procedural details were systematically recorded in the electronic medical record registry, including the operator, nodule or lesion size (short and long diameters), lesion location, lesion characteristics (solid, part-solid, or pure ground-glass opacity [GGO]), the presence of the bronchus sign on chest CT, radial probe positioning after lesion detection, use of adjunctive devices, procedure duration, and any complications encountered during the procedure (11).

### Classification of biopsy results

The results obtained from the TBLB were categorized as malignancy or negative for malignancy. Pathology reports labeled as “negative for malignancy” were further reviewed and the findings classified as specific benign results, non-specific benign results, non-diagnostic results, and atypia (atypical cells). The specific benign results were further subdivided into four groups (Supplementary Table S1): organizing pneumonia (group 1), granuloma (group 2), and fungal infection (group 3). Even in cases of specific benign pathology, the result was considered non-diagnostic if it failed to adequately explain the lung lesion and led to immediate additional invasive procedures.

### Diagnostic definitions

A finding of malignancy on TBLB was considered diagnostic for lung lesions. For non-malignant pathologies, the final diagnosis was determined based on further evaluation, including microbiological cultures (bronchial wash or tissue), response to specific antimicrobial treatments based on chest CT findings, follow-up imaging, or additional invasive procedures such as a second radial EBUS-guided TBLB, percutaneous needle aspiration (PCNA), or surgical resection. The diagnostic yield was calculated as follows, based on three definitions, conservative, intermediate, and liberal, according to previously published guidelines:

1. Conservative diagnostic yield = (Malignant results + Specific benign results)/Total procedures.
2. Intermediate diagnostic yield = (Malignant results + Specific benign results + Non-specific benign results)/Total procedures.
3. Liberal diagnostic yield = (Malignant results + Specific benign results + Non-specific benign results + Atypical + Non-diagnostic results)/Total procedures.

Diagnostic accuracy was calculated using a final reference standard, which classified lesions as malignant or benign. Benign lung lesions were confirmed either by their improvement following specific treatment or by the absence of malignant progression as seen on chest CT after more than 1 year of follow-up. Diagnostic accuracy was defined as the sum of true positives (malignant results) and true negatives (benign results), divided by the total number of procedures. Conservative, intermediate, and liberal diagnostic accuracies were calculated according to the respective definition. For instance, conservative diagnostic accuracy was calculated as: (True positive [malignant] + True negative [specific benign excluding confirmed malignant results])/Total procedures.

### Prediction model for the diagnostic success

A multivariate logistic regression model was used to identify the factors predicting successful lung biopsy. To enhance machine-learning model performance for the diagnostic success, categorical variables were preprocessed using one-hot encoding, while numerical variables were normalized using z-score transformation. Logistic LASSO regression was employed for feature selection. The diagnostic performances of the different models for logistic regression, support vector machine (SVM), and random forest (RF) were evaluated and compared using five-fold cross-validation. Additionally, a multilayer perceptron (MLP) model was developed and subsequently optimized with binary cross-entropy loss and the Adam optimizer. The optimal MLP model was selected based on the epoch with the minimum test loss. Model performance was assessed using the area under the curve (AUC). All statistical analyses and machine-learning processes were conducted using R version 4.3.3 (R Foundation for Statistical Computing, Vienna, Austria) and MATLAB (The MathWorks, Natick, MA). The MLP model was implemented using the open-source Python library TensorFlow (v2.17.0).

## Results

### Patient characteristics

During the study period, a total of 894 R-EBUS-guided TBLB procedures were performed. After excluding 34 repeat procedures in the same patients, 90 procedures performed for rebiopsy to confirm recurrence or for genetic analysis, and 34 patients lost to follow-up within 1 year, a total of 736 patients were included in the final analysis. Malignant lesions were diagnosed in 584 patients and benign lesions in 152 patients based on the final diagnostic outcomes. The prevalence of malignancy was 79.4%. The sizes of the nodules or lung lesions were comparable between the malignant and benign groups (23.0 mm vs. 21.5 mm, *p* = 0.314). The majority of lung lesions were solid (66.2%), followed by part-solid (17.9%), consolidation (7.7%), cavity (5.8%), and pure GGO (2.3%). Most were located in the intermediate zone, with a nearly equal distribution of central and peripheral locations. In terms of the CT-bronchus sign (CT-BS) subclassification (Supplementary Figure S1), classes Ia and Ib comprised 35.6 and 37.9% of results, respectively, followed by Ic (12.8%), IIa (8.8%), IIb (4.6%), and IIc (0.3%); the differences between malignant and benign lesions were not significant. Details of the malignant and benign results are provided in Table 1.

**Table 1:** Demographics.

	Overall	Benign	Malignant	*p* value
Patients (*n*)	736	152	584	
Age, years	68.5 (61.0–75.0)	63.0 (55.5–70.0)	70.0 (62.0–76.0)	<0.001
Sex, male	437 (59.4)	99 (65.1)	338 (57.9)	0.1
Smoking history				0.578
Never smoker	331 (45.0)	68 (44.7)	263 (45.0)	
Ex-smoker	255 (34.6)	57 (37.5)	198 (33.9)	
Current smoker	150 (20.4)	27 (17.8)	123 (21.1)	
Mean size (mm)^*^	22.5 (16.5–31.0)	21.5 (16.0–31.5)	23.0 (17.0–31.0)	0.314
Lobe				0.226
RUL	184 (25.0)	42 (27.6)	142 (24.3)	
RML	71 (9.6)	19 (12.5)	52 (8.9)	
RLL	142 (19.3)	23 (15.1)	119 (20.4)	
LUL	213 (28.9)	38 (25.0)	175 (30.0)	
LLL	126 (17.1)	30 (19.7)	96 (16.4)	
Lesion characteristics				<0.001
Solid	487 (66.2)	94 (61.8)	393 (67.3)	
Part solid >50%	96 (13.0)	10 (6.6)	86 (14.7)	
Part solid <50%	36 (4.9)	4 (2.6)	32 (5.5)	
Pure GGO	17 (2.3)	5 (3.3)	12 (2.1)	
Cavity	43 (5.8)	14 (9.2)	29 (5.0)	
Consolidation	57 (7.7)	25 (16.4)	32 (5.5)	
Lesion location				0.343
Peripheral	162 (22.0)	37 (24.3)	125 (21.4)	
Intermediate	407 (55.3)	87 (57.2)	320 (54.8)	
Central	167 (22.7)	28 (18.4)	139 (23.8)	
Subclass CT classification				0.259
Ia	262 (35.6)	58 (38.8)	203 (34.8)	
Ib	279 (37.9)	53 (34.9)	226 (38.7)	
Ic	94 (12.8)	14 (9.2)	80 (13.7)	
IIa	65 (8.8)	15 (9.9)	50 (8.6)	
IIb	34 (4.6)	11 (7.2)	23 (3.9)	
IIc	2 (0.3)	0 (0.0)	2 (0.3)	
Radial probe position				0.836
Adjacent	108 (14.7)	21 (13.8)	87 (14.9)	
Within	628 (85.3)	131 (86.2)	497 (85.1)	
Procedure time (min)	13.0 (8.0–17.0)	13.0 (9.0–17.0)	12.0 (8.0–18.0)	0.496
Guide sheath use	35 (4.8)	6 (3.9)	29 (5.0)	0.755

Data are reported as median (interquartile range) and number (%). EBUS, endobronchial ultrasound; GGO, ground-glass opacity; LLL, left lower lobe; LUL, left upper lobe; RLL, right lower lobe; RML, right middle lobe; RUL, right upper lobe. The data are presented as *n* (%) or as the median (interquartile range). * Mean size refers to the average of the longest and shortest diameters of the lung lesion.

### Diagnostic performance

**Figure 1:**
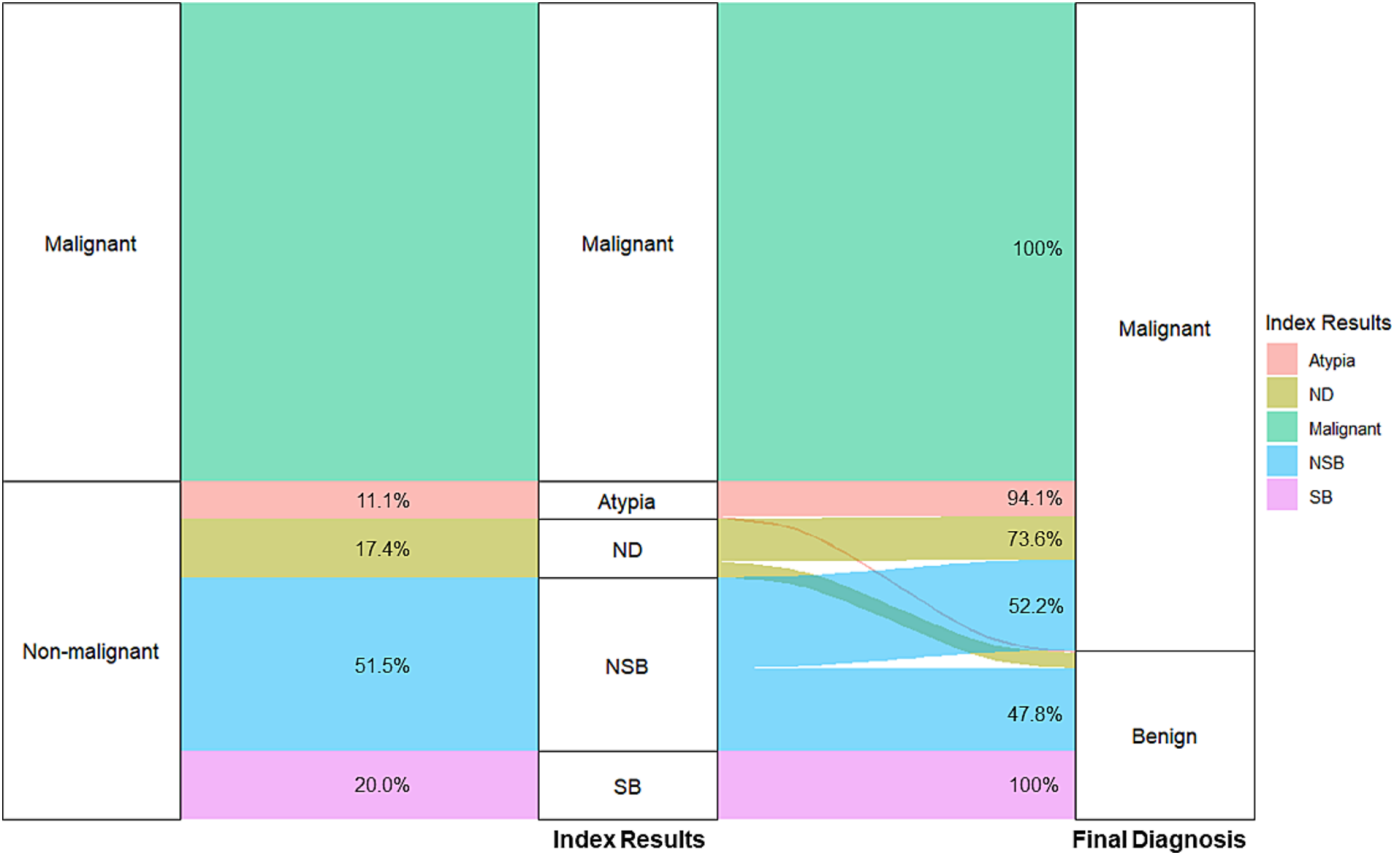
Flow diagram of the process from the index pathology to the final diagnosis. R-EBUS was used to categorize lesions broadly as benign or malignant. The index results provided a further breakdown of non-malignant pathologies. Final diagnoses were confirmed through additional invasive procedures or chest CT follow-up, ensuring accurate differentiation between benign and malignant conditions. CT, computed tomography; R-EBUS, radial endobronchial ultrasound; SB, specific benign; NSB, non-specific benign; ND, non-diagnostic.

**Figure 2:**
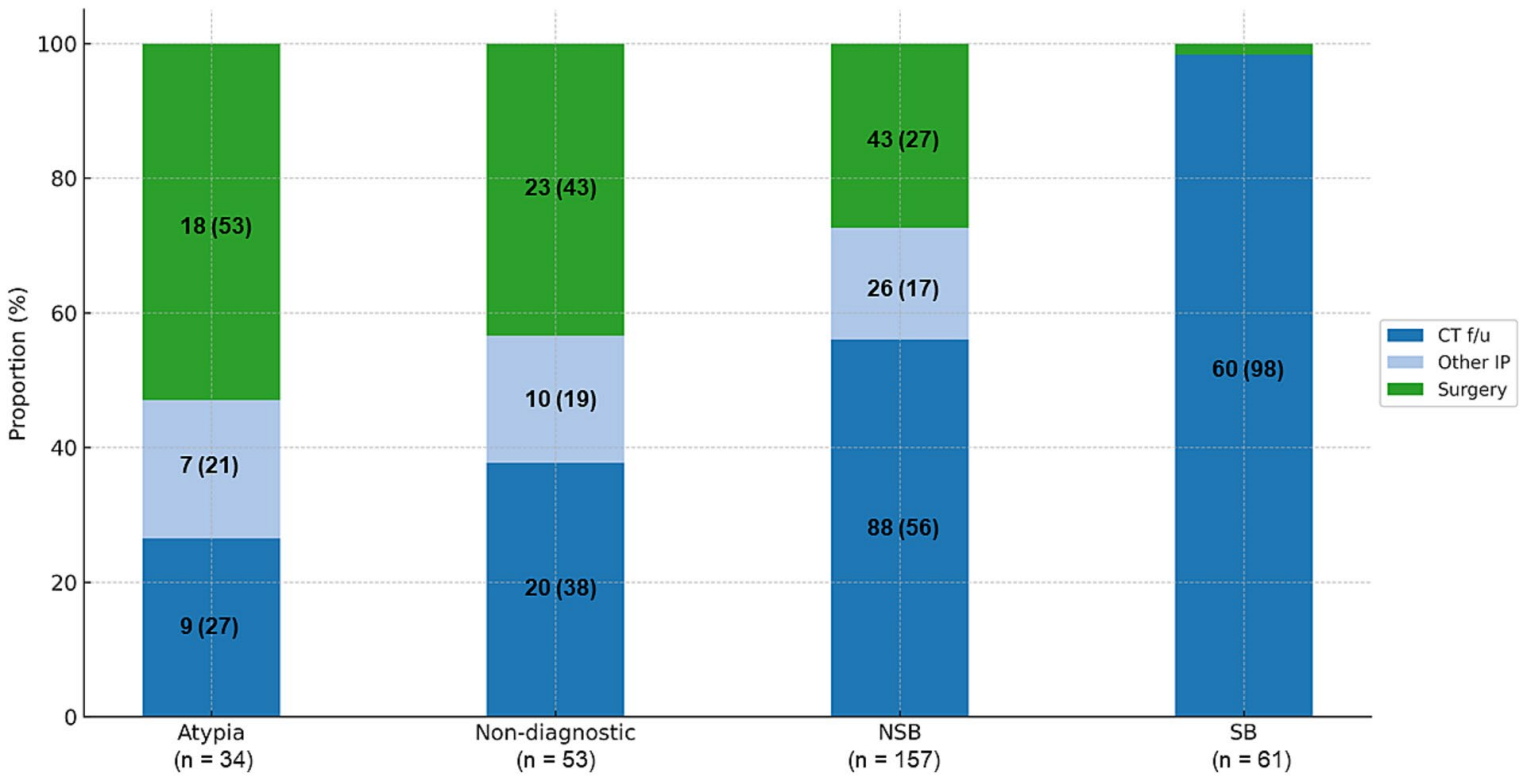
Subsequent diagnostic processes according to index results. Surgery was performed in patients in whom a malignancy was strongly suspected based on the chest CT and positron emission tomography–CT images. Other IPs included percutaneous needle aspiration, EBUS-transbronchial needle aspiration, or additional R-EBUS guided lung biopsy. Data are reported as numbers (%). CT, computed tomography; EBUS, endobronchial ultrasound; R-EBUS, radial endobronchial ultrasound; IP, invasive procedures; SB, specific benign; NSB, non-specific benign.

The pathology results of the 736 patients who underwent R-EBUS-guided TBLB revealed malignancy in 431 (58.6%) and no evidence of malignancy in 305 (41.4%). Non-malignant results were subclassified into four categories: specific benign results (20%), non-specific benign results (51.5%), atypia or atypical cells (11.1%), and non-diagnostic results (17.4%). Five patients with specific benign pathology were also classified as non-diagnostic results, as they underwent immediate invasive procedures. Of these five patients, four were ultimately diagnosed with malignancy, including three cases of organizing pneumonia and one case of granuloma. As shown in Figure 1, benignity was finally confirmed in all patients with specific benign results, 47.8% of those with non-specific benign results, 5.9% of those with atypia, and 26.4% of those with non-diagnostic results. Figure 2 illustrates subsequent diagnostic processes according to index results. Only one patient in the specific benign results required an invasive procedure, which was performed 12 months after the index R-EBUS-guided TBLB as curative surgery for chronic pneumonia. In all other patients with specific benign results, benignity was confirmed through chest CT follow-up. In the non-specific benign results, 56.1% of patients had chest CT follow-up and 27.4% underwent surgery or 16.6% required other invasive procedures. Patients with atypia had higher rates of invasive interventions, with 52.9% requiring surgery, 20.6% receiving other invasive procedures, and 28.1% undergoing chest CT follow-up.

**Figure 3:**
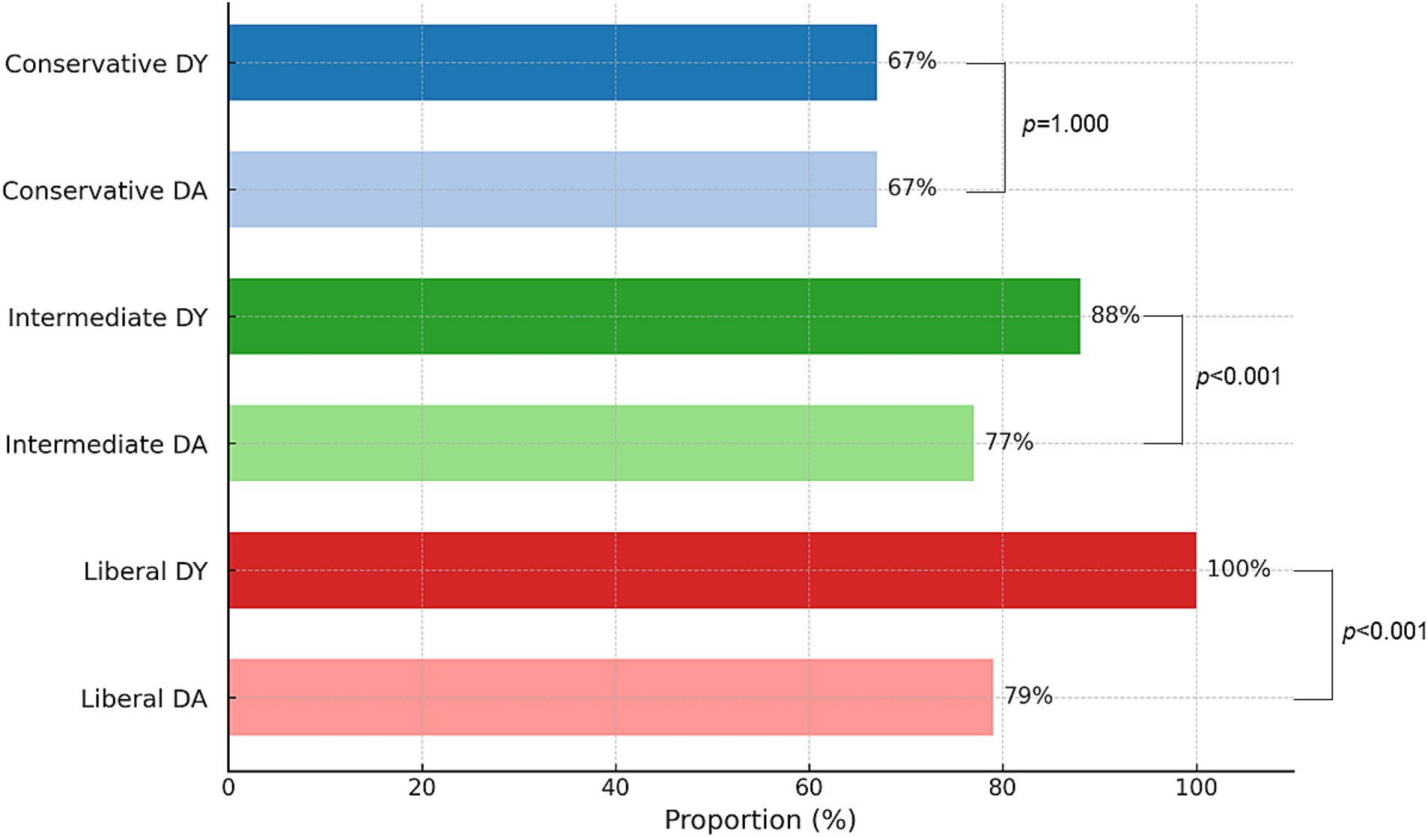
Diagnostic metrics according to conservative, intermediate, and liberal criteria. DY and DA were calculated using predefined methods. The denominator included all patients who underwent R-EBUS-guided lung biopsy. DA, diagnostic accuracy; DY, diagnostic yield.

The overall diagnostic yield and accuracy of R-EBUS-guided lung biopsy were calculated according to pre-defined diagnostic criteria. Diagnostic yield and accuracy were 67 and 67%, respectively, using the conservative definition, 88 and 77% using the intermediate definition, and 100 and 79% using the liberal definition (Figure 3). Table 2 summarizes the diagnostic yields using the conservative definition according to lesion size, lesion characteristics, and CT-BS subclassification. Lesions with a mean diameter of 30–40 mm had a higher diagnostic yield (78.8%), and those < 10 mm had a lower diagnostic yield (45.9%) (*p* < 0.001). The diagnostic yields for solid nodules, cavitary lesions, and consolidation were 69.2, 65.1, and 54.9%, respectively, while for part-solid nodules and pure GGO they were 65.2 and 47.1%, respectively. According to the CT-BS subclassification, diagnostic yields were 69.5% for class Ia and 73.8% for class Ib, but lower for classes Ic, IIa, and IIb (55.3, 55.4, and 44.1%, respectively).

**Table 2:** Conservative diagnostic yields according to nodule size, tumor characteristics, and bronchus classification.

(1) Size						
Nodule size	<10 (*n* = 37)	10–20 (*n* = 254)	20–30 (*n* = 245)	30–40 (*n* = 118)	≥40 (*n* = 82)	*p* value
Conservative DY	45.9%	59.4%	69.8%	78.8%	73.2%	<0.001

(2) Tumor characteristics						
Characteristics	Solid (*n* = 487)	Part solid (*n* = 132)	Pure GGO (*n* = 17)	Cavity (*n* = 43)	Consolidation (*n* = 57)	*p* value
Conservative DY	69.2%	65.2%	47.1%	65.1%	54.9%	0.164

(3) CT subclassification							
Bronchus classification	Ia (*n* = 262)	Ib (*n* = 279)	Ic (*n* = 94)	IIa (*n* = 65)	IIb (*n* = 34)	IIc (*n* = 2)	*p* value
Conservative DY	69.5%	73.8%	55.3%	55.4%	44.1%	50.0%	<0.001

DY, diagnostic yields; GGO, ground-glass opacity.

### Predictive factors for conservative diagnostic success

**Figure 4:**
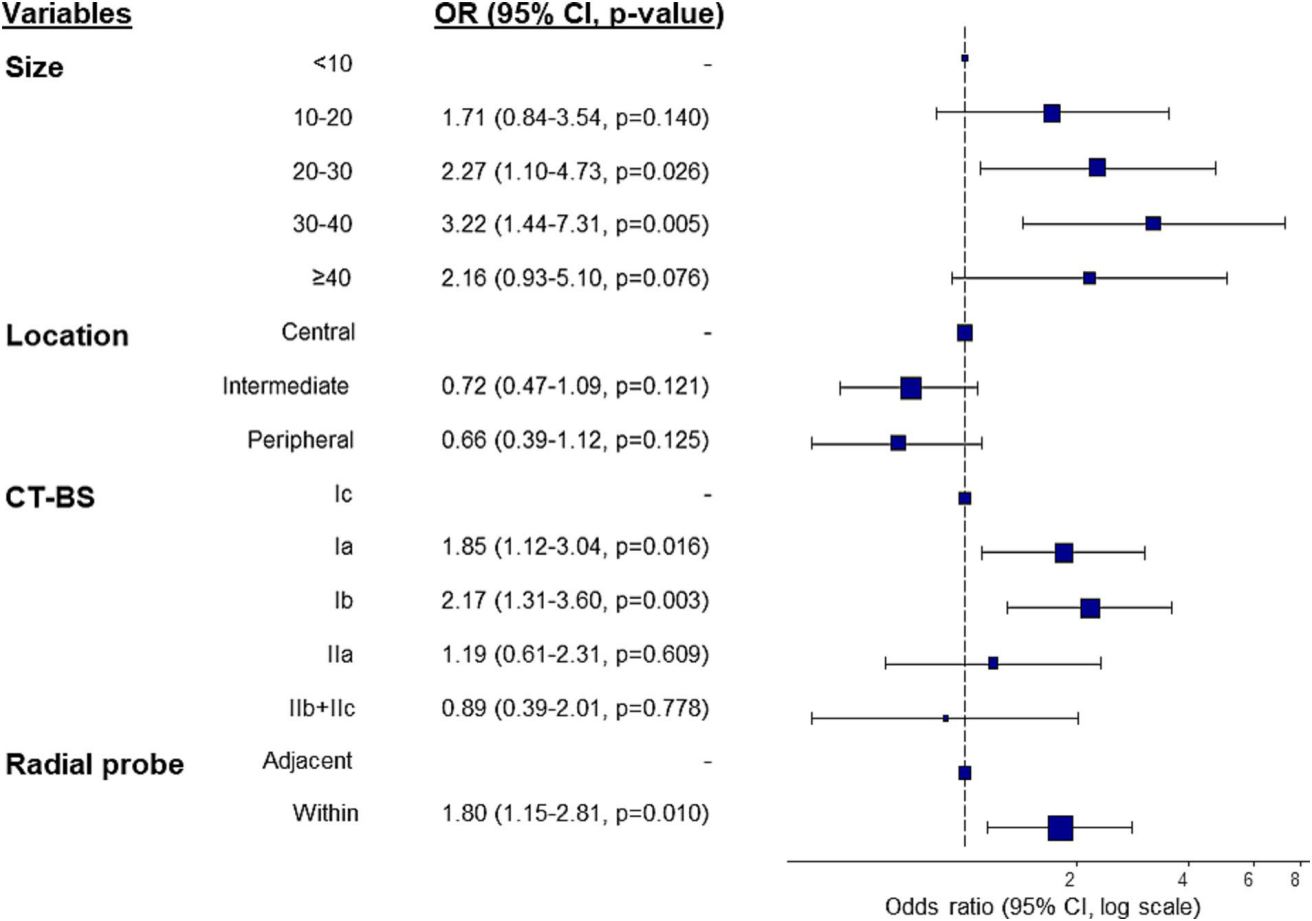
Predictors of conservative diagnostic yield in multivariate logistic regression analysis. CI, confidence interval; CT-BS, computed tomography-bronchus sign.

Univariate and multivariate analyses were conducted to identify the predictive factors associated with diagnostic success based on the conservative definition following R-EBUS-guided TBLB. In the univariate analysis, lung lesions, lesion location, CT-bronchus sign (CT-BS) subclassification and radial probe position were identified as potential predictors of diagnostic success (Supplementary Table S2). These significant variables were included in the multivariate analysis to adjust for confounders and thus determine the independent factors related to diagnostic success. As shown in Figure 4, lesion size, CT-BS sign, and radial EBUS probe position were significantly associated with improved diagnostic success, indicating their importance in predicting outcomes following R-EBUS-guided lung biopsy.

### Machine learning performance using conservative criteria

Our initial hypothesis was that the data of the diagnostic success and failure groups would be linearly separable using machine-learning methods. However, a principal component analysis indicated that distinguishing between these two groups would not be straightforward. The five-fold cross-validated accuracy of the various machine-learning models applied to the testing dataset was as follows: logistic regression: 0.61, SVM: 0.63, RF: 0.64, and MLP: 0.59. The AUC was highest for the SVM and RF models (Table 3). As expected, the predicted scores showed significant overlap between the diagnostic success and non-diagnostic groups, indicating that the models were unable to distinguish clearly between them, such that the overall predictive performance was accordingly reduced (Supplementary Figure S2).

**Table 3:** Machine-learning model performance with conservative criteria (five-fold cross-validation average).

	Accuracy	Sensitivity	Specificity	AUC
Logistic				
Training	0.6496	0.6489	0.6507	0.6907
Test	0.6061	0.6117	0.5942	0.6309
SVM				
Training	0.6715	0.7790	0.4551	0.6303
Test	0.6331	0.7434	0.4093	0.5825
RF (128-tree)				
Training	0.8390	0.9736	0.5680	0.8824
Test	0.6427	0.8515	0.2208	0.6049
MLP				
Training	0.7106	0.7119	0.7071	0.7657
Test	0.5883	0.6292	0.5075	0.6065

SVM, support vector machine; RF, random forest; MLP, multilayer perception. Model performance evaluated at best validation loss. Predicted label decision using Youden’s J index of the ROC curve.

## Discussion

A previous study found that the conservative diagnostic yield was the most effective metric for evaluating the performance of navigation bronchoscopy in diagnosing lung lesions (10). To determine whether the conservative diagnostic yield can be used to assess the performance of R-EBUS-guided lung biopsy reliably, in this study it was compared with diagnostic accuracy, as a traditional metric. In addition, the potential of the conservative diagnostic yield as a standard measure for evaluating guided bronchoscopy techniques was explored. In epidemiology, diagnostic accuracy is widely used to compare the performance of different diagnostic tools in diagnosing specific diseases (12). However, diagnosis of lung nodules presents unique challenges due to the distinct characteristics of benign and malignant lesions. For benign pathologies, it is important to classify the findings into subcategories, such as specific benign, non-specific benign, or normal lung tissue, so that the reliability of the pathology results can be accurately assessed and appropriate clinical decisions then made. A precise classification helps to ensure that the results are trustworthy and useful for guiding further medical actions (13).

The category specific benign pathology can be broadly classified into four subcategories highly indicative of inflammation, i.e., organizing pneumonia, granuloma, and fungal infections, as well as a benign tumor category. These subcategories are clinically significant because they allow physicians to make informed decisions on whether to proceed with further invasive procedures to investigate the nature of a lung nodule or to treat the lesion based on the index pathology results. As most non-specific benign pathologies involve inflammation and fibrosis, it is crucial to determine whether they adequately explain the lung nodule or consolidation.

A comparison of diagnostic yield and diagnostic accuracy must take into account that the latter requires a minimum follow-up time to confirm true-negative results in patients in whom the index pathology shows no malignancy (14, 15). A major limitation of using diagnostic accuracy as a performance metric is that it can overestimate diagnostic performance, even when the target lesion was not accurately reached but the result was later confirmed as benign. Another limitation is the time-consuming nature of determining diagnostic accuracy, as it requires follow-up periods of at least 1–2 years, making it less than ideal as a patient-centered outcome metric. Our data demonstrate the value of using specific benign results as a meaningful numerator in the calculation of diagnostic yield. Benignity was confirmed in all cases with specific benign results during the follow-up period. By contrast, non-specific benign results, atypical cells, and non-diagnostic results were confirmed as benign in only 47.8, 5.9, and 26.4% of patients, respectively. Importantly, when atypical cells were identified at the index pathology exam, most of the lesions were later determined to be malignant, underscoring the need for aggressive management in such cases to elucidate fully the nature of the pathology (16).

This study also evaluated diagnostic performance using three different definitions of diagnostic yield—conservative, intermediate, and liberal—and compared them with diagnostic accuracy to identify the definition most closely aligned with diagnostic accuracy, thereby providing a meaningful measure of procedure success. Previous definitions of conservative, intermediate, and liberal diagnostic yields were based on how the numerator is defined in relation to the total number of procedures performed (10, 17). The inclusion of specific benign or non-specific benign results, in addition to malignant results, in the numerator was shown ultimately to influence patient outcome.

To assess the impact of diagnostic yield on clinical decision-making, we reviewed cases in which additional invasive procedures or surgical resections were performed, given our knowledge of the index pathology results. Only one patient in the specific benign results required an invasive procedure—surgery for the management of chronic pneumonia. However, for patients with non-specific benign results, the rates of further invasive procedures and surgical resection increased to 16.6 and 27.4%, respectively. The surgical resection rates among patients with atypical cells were 52.9%. These findings emphasize the impact of diagnostic yield on patient decision-making and the need for further confirmation in patients with non-specific benign results, atypical cells, or non-diagnostic results, in contrast to those with specific benign results, which are more likely to represent a definitive diagnosis. Diagnostic accuracy and diagnostic yield were identical under the conservative definition; however, the intermediate and liberal definitions showed significant discrepancies, consistent with previous studies. The results validate the use of conservative diagnostic yield as a performance metric with favorable implications for decision-making. Nonetheless, it is important to recognize that diagnostic accuracy remains a valuable metric, particularly in research settings and when long-term follow-up data are available. Each metric offers distinct advantages, and both should be interpreted in light of the specific clinical or investigative context.

Identification of the factors predicting diagnostic success can contribute to better outcomes in interpretations of lung biopsies when the conservative diagnostic yield definition is used. This study identified lesion size, CT-BS, and radial probe position within the lesion as predictors of diagnostic success. Previous studies have shown the importance of the bronchus sign as a predictive factor in bronchoscopy (18–20). The bronchus sign is often classified based on its position relative to the lesion (within or adjacent) (21). In a recently proposed classification system, the bronchus sign is divided into five subgroups (22) to inform bronchoscopists better about whether to proceed with bronchoscopy or opt for other invasive diagnostic techniques. This subclassification, along with lesion size, location, and characteristics, was considered in our analysis of the factors predicting diagnostic success using conservative diagnostic yield as the definition.

Diagnostic yield did not significantly differ based on lesion characteristics, such as solid nodules, part-solid nodules, pure GGO, cavitary lesions, and consolidation. However, according to the CT-BS, diagnostic yields were higher in classes Ia and Ib than in the other subclasses. Class Ib, in which the bronchus enters the lesion but does not penetrate the nodule, closely aligns with the traditional “within the lesion” sign and had a diagnostic success rate of 73.8%. By contrast, the success rates for adjacent bronchus signs (classes Ic, IIa, IIb) were lower, ranging from 44 to 55%. Importantly, the diagnostic success rate of class Ic, in which the bronchus penetrates the lesion like a tunnel, was similar to that of class IIa, which indicates close contact with the lesion even though it may appear as “within the lesion.”

Multivariate logistic regression identified lesion size, CT-BS (classes Ia and Ib), and radial probe position within the lesion as independent predictive factors for successful diagnosis based on the conservative diagnostic yield. Additionally, the prediction of diagnostic outcome based on conservative criteria using machine-learning models was explored by evaluating the performance of logistic regression, SVM, RF, and MLP. However, although they identified several predictive factors, the performance of all of these machine-learning models was unsatisfactory. An analysis of the distribution of the predicted scores showed significant overlap between the diagnostic success and failure groups, indicating that it would be impractical to use a simple probability cutoff to predict outcomes. Further improvement of machine-learning models is needed to enhance their prediction accuracy, particularly by incorporating 3D geometric features of the bronchial pathway, which were identified in our previous study (23) as significant predictors of navigation success but were not included in the current model.

This study was designed to understand better how the conservative diagnostic yield can be effectively used in clinical practice to improve the standardization and accuracy of lung biopsy techniques. However, the limitations of this study must also be considered. First, despite a large sample size of 736 patients, our results may not be generalizable to all patient populations due to the single-center design of the study and its focus solely on R-EBUS-guided lung biopsy. Additionally, the retrospective nature of the study introduces potential biases related to patient selection and data collection. The classification of pathology results into diagnostic categories was performed retrospectively and was not blinded to follow-up outcomes or final diagnoses, which may have introduced bias in diagnostic categorization. In addition, exclusion of a subset of patients who were lost to follow-up within 1 year may have led to potential bias, particularly if patients with specific nodule characteristics were more prone to being lost. Prospective studies are needed to validate our findings and minimize bias.

Nonetheless, our study underscores the importance of using standardized metrics, such as conservative diagnostic yield, to evaluate the performance of lung biopsy techniques, as they can have a significant impact on patient management and outcomes. The validation of our findings awaits studies with a prospective design while development of more robust predictive models will minimize unnecessary procedures and improve clinical decision-making.

In conclusion, our study validated the use of the conservative definition of diagnostic yield as a reliable diagnostic endpoint for evaluating the performance of guided bronchoscopy. This definition could serve as a time-saving standard in prospective studies comparing the diagnostic effectiveness of various navigation devices.

## Data Availability

The raw data supporting the conclusions of this article will be made available by the authors, without undue reservation.
