# Supplementary material for: Evaluating diagnostic yield and accuracy as key performance metrics in pulmonary lung lesions
Source: Front Med (Lausanne). 2025 May 7;12:1572779. doi: 10.3389/fmed.2025.1572779 (PMC12092437; doi:10.3389/fmed.2025.1572779)
Supplement: Supplementary file 1 [file Table_1.docx]

**Table S1** Classification of benign pathology.

| **Specific benign pathology** | **Nonspecific benign pathology** | **Nondiagnostic pathology** |
| --- | --- | --- |
| **Organizing pneumonia category** | Chronic inflammation and fibrosis  Mild stromal fibrosis  Hyperplastic pneumocytes  Parenchymal dense fibrosis  Intra-alveolar aggregation of macrophages  Anthracofibrosis  Acute inflammation and fibrosis  Focal inflammation with hemorrhage  Stromal fibrosis  Intraalveolar many mucin-containing macrophages  Acellular extensive necrosis  Interstitial lymphocytic infiltration with loose myxoid polyp  Necrosis with microabscess  Neutrophilic aggregation, abscess  Dense lymphoplasma cell infiltration  A cellular amorphous eosinophilic tissue  Lymphoplasmacytic infiltration  Neutrophilic abscess or inflammation  Eosinophilic infiltration | A few activated pneumocytes and macrophages  Lung parenchyma with intraalveolar hemorrhage  Peribronchial tissue only  Emphysema with anthracosis  Bronchial mucosa showing inflammation  Small fragments of lung tissue |
| Organizing pneumonia  Organizing pneumonia with some fibrin  Organizing pneumonia, Focal microabscess  Organizing pneumonia with interstitial chronic inflammation  Organizing pneumonia with hemorrhage  Organizing pneumonia with some hemorrhage |  |  |
| **Granuloma category** |  |  |
| Granuloma  Granulomatous inflammation  Granulomatous pneumonia  Several non-necrotic granulomas  Chronic inflammation with granulomas and necrosis  Multiple granulomas  Granuloma with chronic inflammation  Caseating granuloma  Necrotic granuloma |  |  |
| **Fungus category** |  |  |
| Fungal ball, morphologically aspergillus species  Cryptococcal granuloma  Fungal hyphae with necrosis |  |  |
|  |  |  |
|  |  |  |

**Table s2**  Univariate logistic analysis for the TBLB success with the conservative definition

| **Characteristic** | **N** | **OR1** | **95% CI1** | ***p*-value** |
| --- | --- | --- | --- | --- |
| Smoking | 736 |  |  |  |
| Current |  | — | — |  |
| Ex-smoker |  | 1.08 | 0.71, 1.64 | 0.7 |
| Never smoker |  | 1.34 | 0.89, 2.01 | 0.2 |
| Lobe | 736 |  |  |  |
| LLL |  | — | — |  |
| LUL |  | 1.47 | 0.92, 2.33 | 0.11 |
| RLL |  | 1.24 | 0.75, 2.06 | 0.4 |
| RML |  | 1.57 | 0.84, 2.99 | 0.2 |
| RUL |  | 1.10 | 0.69, 1.76 | 0.7 |
| Size (mm) | 736 |  |  |  |
| <10 |  | — | — |  |
| >=40 |  | 3.21 | 1.44, 7.32 | 0.005 |
| 10-20 |  | 1.72 | 0.86, 3.48 | 0.12 |
| 20-30 |  | 2.72 | 1.35, 5.54 | 0.005 |
| 30-40 |  | 4.38 | 2.01, 9.70 | <0.001 |
| Lesion characteristics | 736 |  |  |  |
| Solid |  | — | — |  |
| Part solid > 50% |  | 0.93 | 0.59, 1.51 | 0.8 |
| Part solid < 50% |  | 0.62 | 0.31, 1.26 | 0.2 |
| Pure GGO |  | 0.40 | 0.15, 1.05 | 0.061 |
| Consolidation |  | 0.61 | 0.35, 1.08 | 0.086 |
| Cavity |  | 0.83 | 0.44, 1.64 | 0.6 |
| Lesion location | 736 |  |  |  |
| Central |  | — | — |  |
| Intermediate |  | 0.69 | 0.46, 1.02 | 0.069 |
| Peripheral |  | 0.59 | 0.37, 0.94 | 0.028 |
| Radial probe position | 736 |  |  |  |
| Adjacent |  | — | — |  |
| Within |  | 2.41 | 1.59, 3.65 | <0.001 |
| Subclass CT classification | 736 |  |  |  |
| Ia |  | — | — |  |
| Ib |  | 1.24 | 0.85, 1.81 | 0.3 |
| Ic |  | 0.54 | 0.34, 0.88 | 0.014 |
| IIa |  | 0.55 | 0.31, 0.95 | 0.032 |
| IIb+IIc |  | 0.35 | 0.17, 0.71 | 0.004 |

Abbreviations: 1OR, Odds Ratio; CI, Confidence Interval.

**Figure 1** Chest CT-Bronchus subclassification.


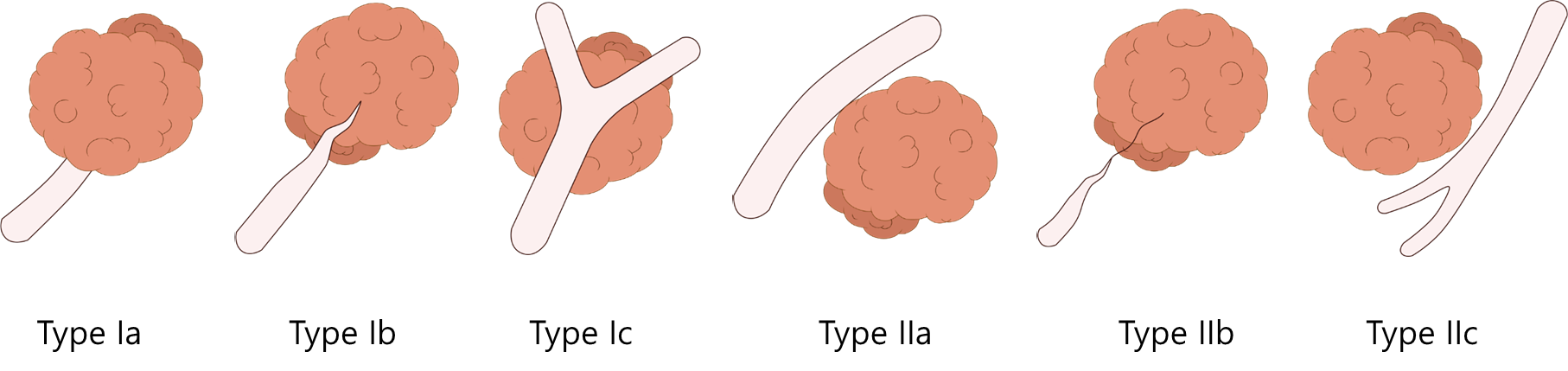


**Figure 2** PCA and prediction score.


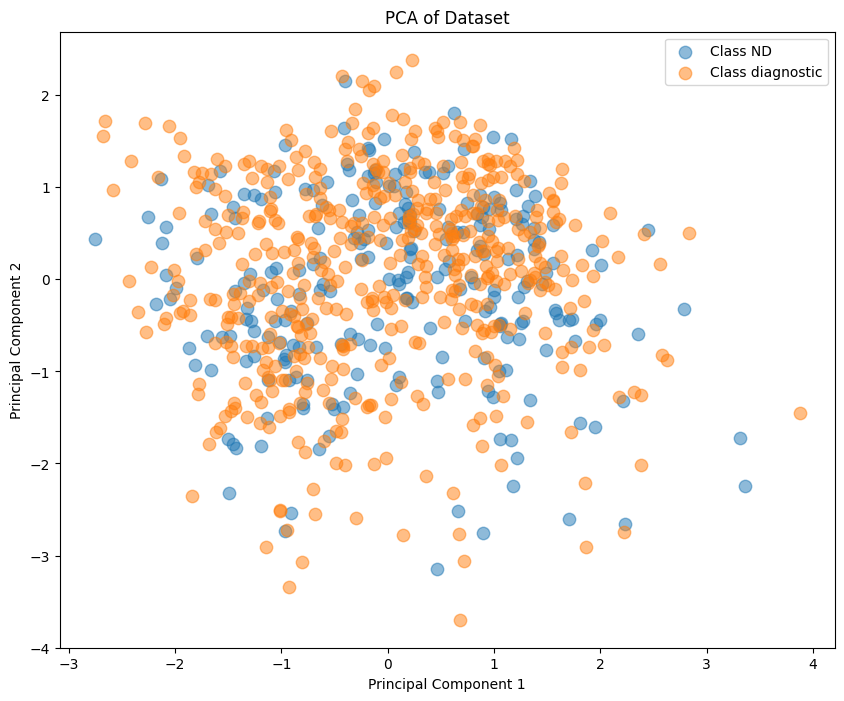


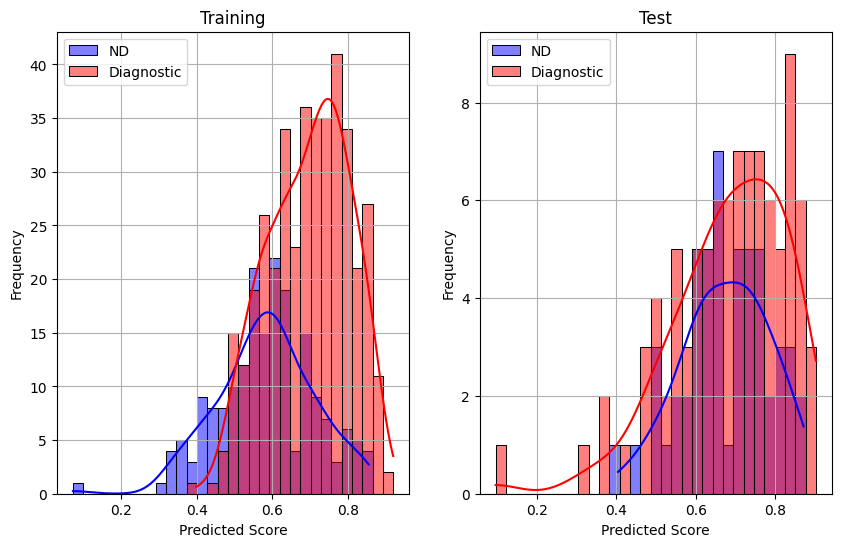


Predicted score distribution of last fold (Fold 5)
