# Supplementary figures and images for: Evaluating diagnostic yield and accuracy as key performance metrics in pulmonary lung lesions
Source: Front Med (Lausanne). 2025 May 7;12:1572779. doi: 10.3389/fmed.2025.1572779 (PMC12092437; doi:10.3389/fmed.2025.1572779)

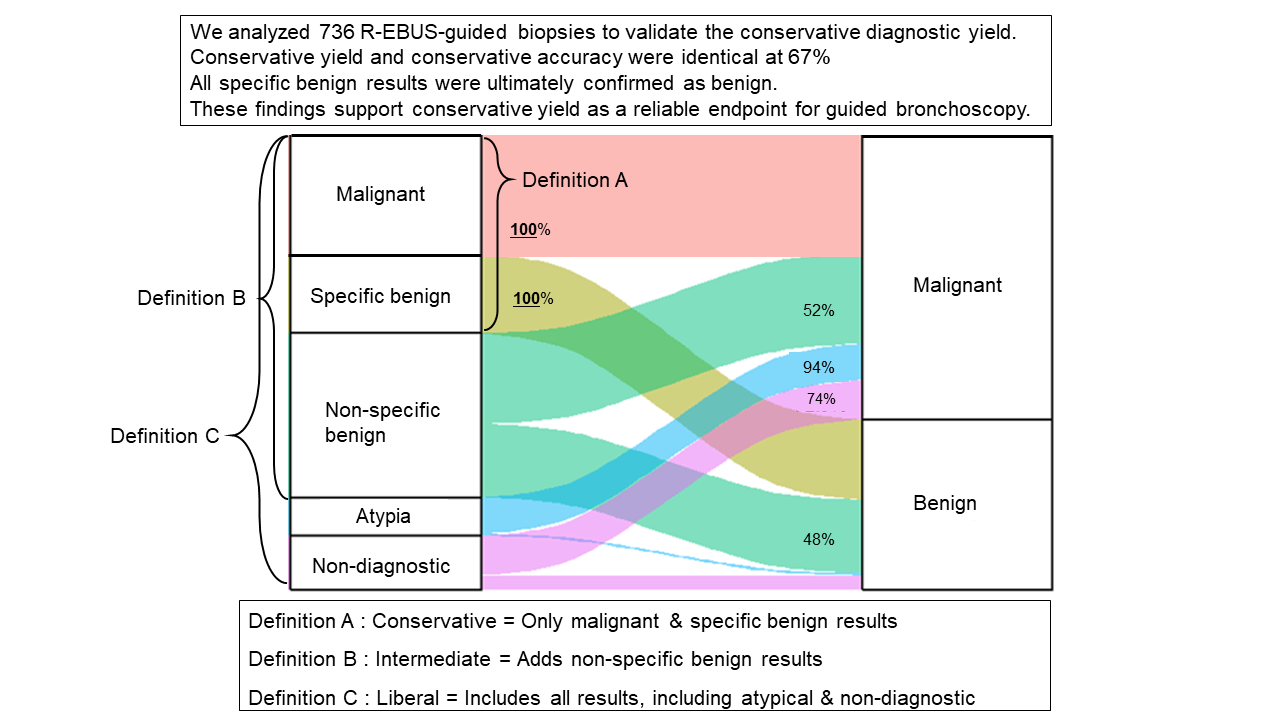

Supplement: Supplementary file 2 [file Image_1.tif]
